# Supplementary material for: Inhibition of Stat3‐mediated astrogliosis ameliorates pathology in an Alzheimer's disease model
Source: EMBO Mol Med. 2019 Jan 7;11(2):e9665. doi: 10.15252/emmm.201809665 (PMC6365929; doi:10.15252/emmm.201809665)
Supplement: Supplementary file 1 — Expanded View Figures PDF [file EMMM-11-e9665-s001.pdf]

Expanded View Figures

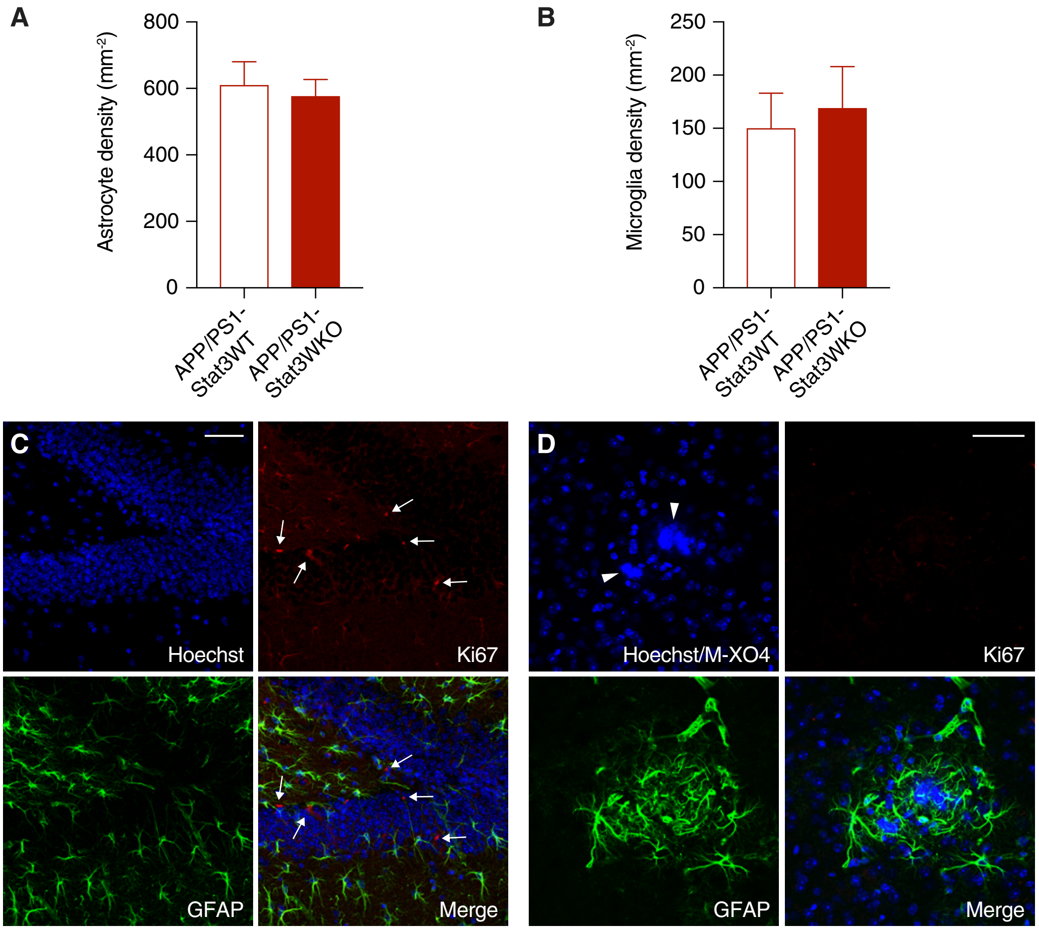

Figure EV1. No evidence for proliferation of near-plaque reactive glia.

A, B The total density of astrocytes and microglia remained unchanged in APP/PS1-Stat3KO compared to APP/PS1-Stat3WT mice (Mann–Whitney test for both comparisons; APP/PS1-Stat3WT, n = 8 (three females and five males) mice; APP/PS1-Stat3KO, n = 8 (five females and three males) mice; age, 8–9 months).

C, D Using an anti-Ki67 antibody as a marker for cellular proliferation, we detected Ki67-positive cells (arrows) in the hippocampal dentate gyrus as a positive control. However, no Ki67 signal was detected around plaques (marked by arrowheads) of either APP/PS1-Stat3KO or APP/PS1-Stat3WT mice, indicating little-to-no glial proliferation (scale bars, 50 μm; same mice as in A and B).

Data information: Data are represented as mean ± SEM.

© 2018 The Authors

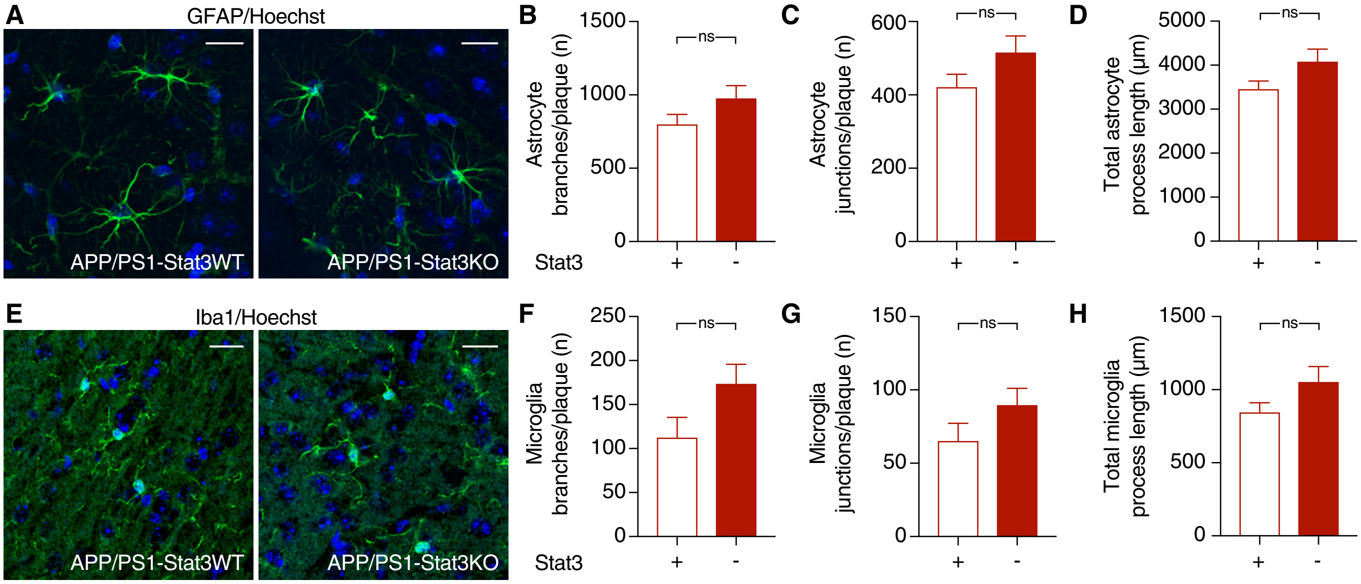

Figure EV2. No significant morphological changes in glial cells remote from plaques.

A–D The morphology of hippocampal astrocytes remote from plaques was largely unaltered in APP/PS1-Stat3KO compared to APP/PS1-Stat3WT mice.

E–H Similarly, the morphology of hippocampal microglia remote from plaques was also similar in APP/PS1-Stat3KO compared to APP/PS1-Stat3WT mice.

Data information: Data are represented as mean ± SEM. Mann–Whitney test for all comparisons; scale bars, 20 µm; APP/PS1-Stat3KO, *n* = 10 (four females and six males) mice; APP/PS1-Stat3WT, *n* = 6 (three females and three males) mice; age, 8–9 months.

© 2018 The Authors

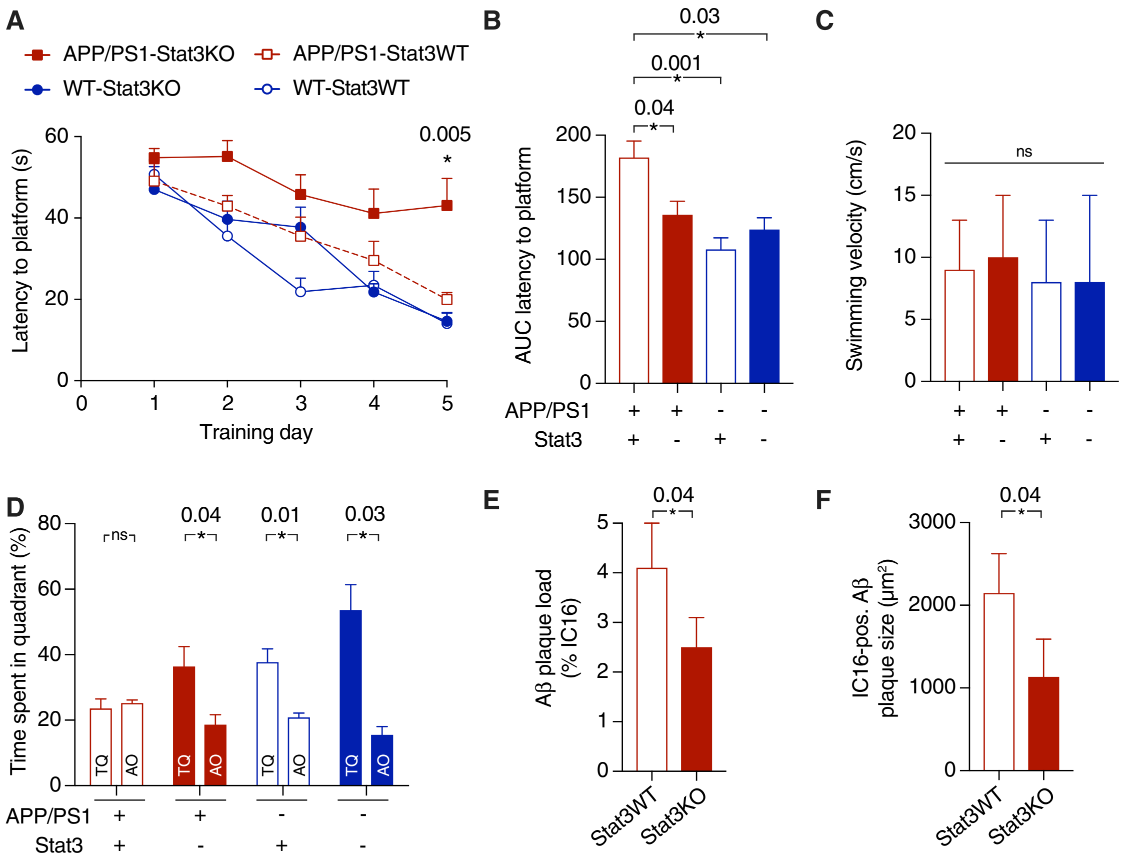

Figure EV3. Persistence of cognitive protection and reduced amyloid pathology in late-stage APP/PS1-Stat3KO mice.

A Spatial learning and memory were assessed in the Morris Water Maze. APP/PS1-Stat3KO mice showed faster latencies to reach the hidden platform compared with APP/PS1-Stat3WT on day 5, but were similar to WT-Stat3WT and WT-Stat3KO mice (*$P < 0.05$, two-way repeated-measures ANOVA followed by Bonferroni post hoc test; *P*-value for APP/PS1-Stat3KO versus APP/PS1-Stat3WT mice).

B, C The area under the curve (AUC) for the latency to reach the hidden platform was similar in APP/PS1-Stat3WT compared to WT-Stat3WT and WT-Stat3KO mice, but significantly higher in APP/PS1-Stat3KO mice (*$P < 0.05$, Kruskal–Wallis test followed by Dunn's multiple comparisons test). The swimming velocity was similar in all groups (Kruskal–Wallis test followed by Dunn's multiple comparisons test).

D In the probe trial, APP/PS1-Stat3KO, WT-Stat3WT, and WT-Stat3KO mice spent significantly more time in the target quadrant (TQ) compared to the mean of all other quadrants (AO), whereas APP/PS1-Stat3WT spent equal times in the target and all other quadrants (*$P < 0.05$, Wilcoxon matched-pairs signed rank test for each comparison).

E, F Plaque load and plaque size were reduced in APP/PS1-Stat3KO compared to APP/PS1-Stat3WT mice (*$P < 0.05$, Mann–Whitney test for both comparisons; same mice as in A–D).

Data information: Data are represented as mean ± SEM. WT-Stat3WT, $n = 12$ (6 females and 6 males) mice; WT-Stat3KO, $n = 10$ (4 females and 6 males) mice; APP/PS1-Stat3WT, $n = 12$ (seven females and five males) mice; APP/PS1-Stat3KO, $n = 12$ (eight females and four males) mice; age, 13–14 months.

© 2018 The Authors
