# Supplementary material for: Inhibition of Stat3‐mediated astrogliosis ameliorates pathology in an Alzheimer's disease model
Source: EMBO Mol Med. 2019 Jan 7;11(2):e9665. doi: 10.15252/emmm.201809665 (PMC6365929; doi:10.15252/emmm.201809665)
Supplement: Supplementary file 2 — Movie EV1 [file EMMM-11-e9665-s002.zip › EMM-2018-09665_MovieEV1/Legend.docx]

**EV Movie legends**

**Movie EV1**

Example of fibrillar Aβ (stained with methoxy-XO4 (blue) and IC16 antibody (red)) engulfed by microglia (Iba1, green). The movie shows the original 3D-reconstructed confocal z-stack and surface-rendered channels.
