# Supplementary figures and images for: Inhibition of Stat3‐mediated astrogliosis ameliorates pathology in an Alzheimer's disease model
Source: EMBO Mol Med. 2019 Jan 7;11(2):e9665. doi: 10.15252/emmm.201809665 (PMC6365929; doi:10.15252/emmm.201809665)

Figure 3 Source Data

MW marker

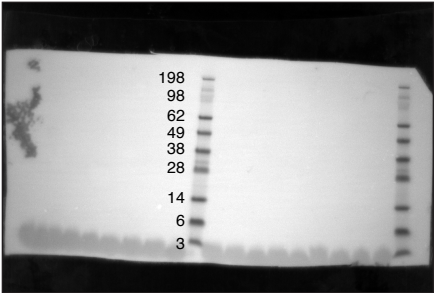

APP

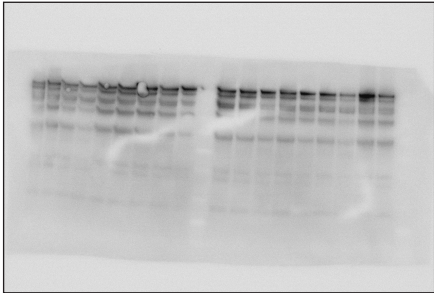

$\beta$ -Actin

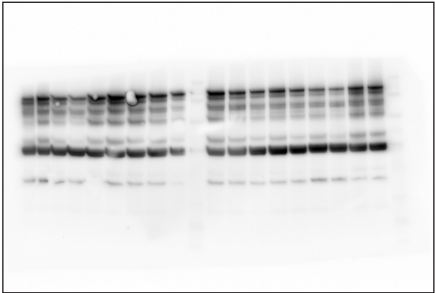

MW marker

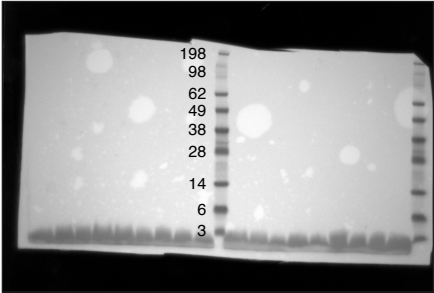

CTF

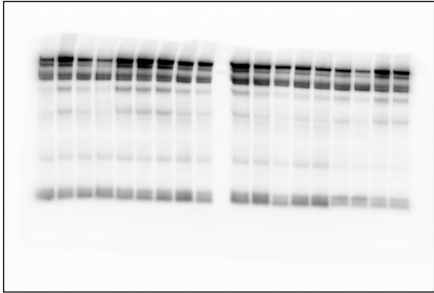

$\beta$ -Actin

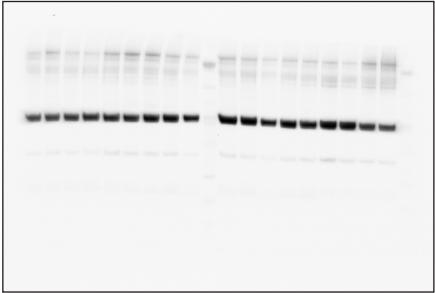

Supplement: Supplementary file 5 — Source Data for Figure 3 [file EMMM-11-e9665-s004.pdf]
