# Supplementary material for: Inhibition of Stat3‐mediated astrogliosis ameliorates pathology in an Alzheimer's disease model
Source: EMBO Mol Med. 2019 Jan 7;11(2):e9665. doi: 10.15252/emmm.201809665 (PMC6365929; doi:10.15252/emmm.201809665)
Supplement: Supplementary file 6 — Source Data for Figure 4 [file EMMM-11-e9665-s005.pdf]

Figure 4 Source Data

MW marker

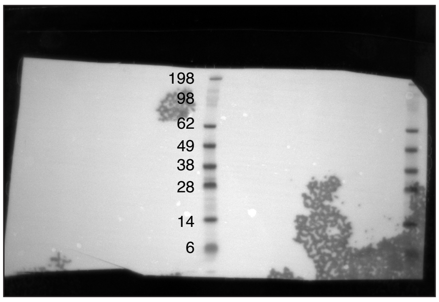

Neprilysin

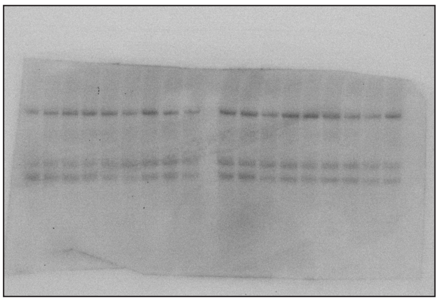

β-Actin

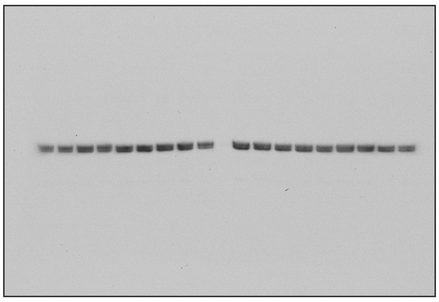

MW marker

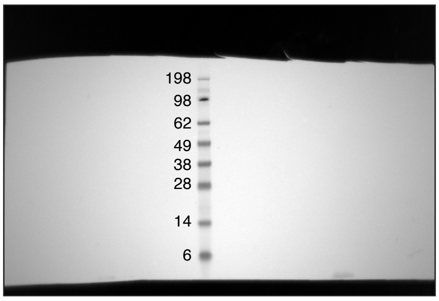

ApoE

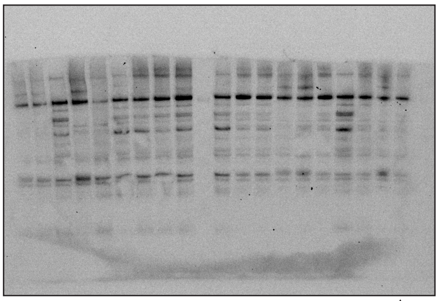

ref. protein

β-Actin

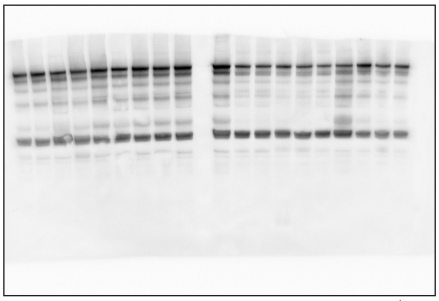

ref. protein

CD36 + MW marker

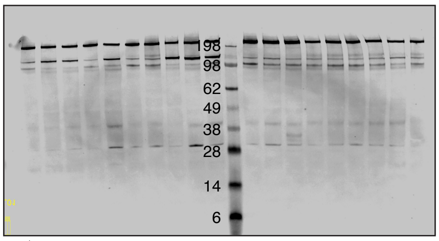

ref. protein

MW marker (β-Actin)

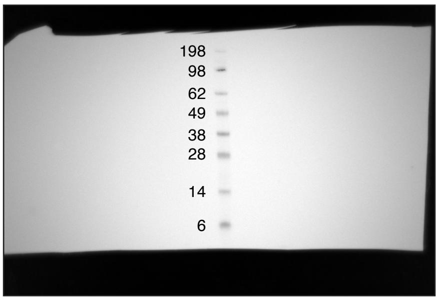

β-Actin

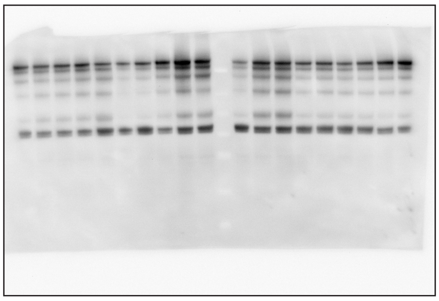

ref. protein

MW marker

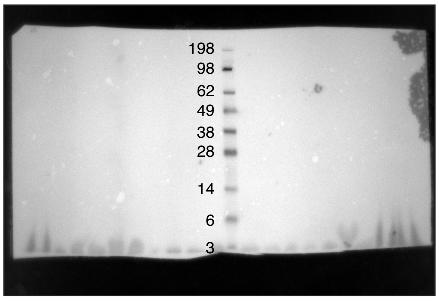

TREM2

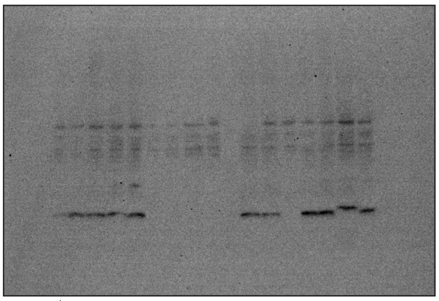

ref. protein

β-Actin

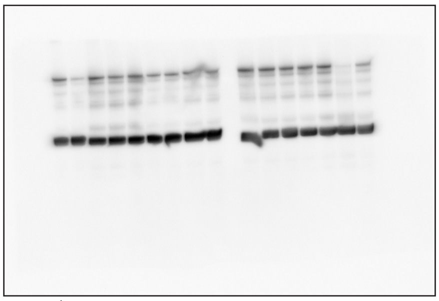

ref. protein
